# Impact of *Rumex nepalensis* on Performance, Blood Markers, Immunity, Intestinal Microbiology and Histomorphology in Broiler Chicken

**DOI:** 10.3390/vetsci11100463

**Published:** 2024-10-01

**Authors:** Mohammad T. Banday, Manzoor A. Wani, Sarah I. Othman, Hassan A. Rudayni, Ahmed A. Allam, Mohammad Y. Alshahrani, Essam H. Ibrahim, Showkat Nabi, Sheikh Adil

**Affiliations:** 1 Division of Livestock Production and Management, Faculty of Veterinary Sciences and Animal Husbandry, Sher-e-Kashmir University of Agricultural Sciences and Technology-Kashmir, Shuhama 190006, India; 2 Department of Biology, College of Science, Princess Nourah bint Abdulrahman University, Riyadh 11671, Saudi Arabia; 3 Department of Biology, College of Science, Imam Mohammad Ibn Saud Islamic University, Riyadh 11623, Saudi Arabia; 4 Department of Zoology, Faculty of Science, Beni-Suef University, Beni-Suef 65211, Egypt; 5 Department of Clinical Laboratory Sciences, College of Applied Medical Sciences, King Khalid University, Abha 61421, Saudi Arabia; 6 Biology Department, Faculty of Science, King Khalid University, Abha 61413, Saudi Arabia; 7 Blood Products Quality Control and Research Department, National Organization for Research and Control of Biologicals, Cairo 12611, Egypt; 8 Division of Veterinary Medicine, Faculty of Veterinary Sciences and Animal Husbandry, Sher-e-Kashmir University of Agricultural Sciences and Technology-Kashmir, Shuhama 190006, India

**Keywords:** broiler chicken, growth, histomorphology, immunology, microbiology, phytogenic, *Rumex*

## Abstract

**Simple Summary:**

The study evaluated the effects of *Rumex nepalensis* leaf powder (RNL) as a natural feed additive in broiler chickens. RNL @ 10 g/kg diet improved the body weight gain and feed conversion ratio of broilers. Total serum cholesterol was decreased when RNL was added @ 5 and 10 g/kg diet. Immunity of birds was better as depicted by increase in the serum levels of immunoglobulin G and immunoglobulin M levels in RNL supplemented groups. RNL @ 10 g/kg diet was found effective in enhancing the antioxidant enzyme status of birds in terms of increased serum superoxide dismutase and decreased malondialdehyde levels. Gut health of broilers improved by RNL as recorded by favorable results in cecal microbiology and intestinal histomorphology. Thus, RNL is a promising natural feed additive for improving the growth, immuno-antioxidant status and gut health of broiler chicken.

**Abstract:**

The study investigated the impact of utilizing *Rumex nepalensis* leaf powder (RNL) as a phytogenic feed additive on performance, blood markers, intestinal microbiology and histomorphology in broiler chicken. One hundred eighty day-old Cobb broiler chicks were randomly divided into four treatment groups having three replicates with fifteen birds each. Four iso-caloric and iso-nitrogenous diets primarily based on maize–soybean were formulated, viz., CN (Control)—fed basal diet only; RNL_2.5_ (basal diet + 2.5 g/kg RNL); RNL_5_ (basal diet + 5 g/kg RNL); and RNL_10_ (basal diet + 10 g/kg RNL). The results revealed a significant (*p* < 0.05) increase in body weight gain and feed conversion ratio in dietary treatments compared to CN with best values in RNL_10_ followed by RNL_5_. The blood markers like glucose, total protein, creatinine, alanine transaminase (ALT) and aspartate transaminase (AST) showed no significance (*p* > 0.05) among all the treatments, however total cholesterol significantly (*p* < 0.05) decreased in RNL_5_ and RNL_10_ as against CN. Regarding immune parameters, immunoglobulin G (IgG) and immunoglobulin M (IgM) levels significantly (*p* < 0.05) enhanced in RNL_5_ and RNL_10_. Antioxidant enzyme status showed that superoxide dismutase (SOD) increased and malondialdehyde (MDA) decreased significantly (*p* < 0.05) in RNL_10_ compared to CN. Gut health in terms of cecal microbiology and histomorphology of duodenum and jejunum were altered by inclusion of RNL in the broiler diet. A significant decrease (*p* < 0.05) in coliform count was recorded by incorporation of dietary treatments with highest reduction in RNL_10_. *Lactobacillus* count and total viable count did not vary significantly (*p* > 0.05) among dietary treatments and CN. Duodenal and jejunal villus height and villus height/crypt depth ratio were significantly (*p* < 0.05) increased in RNL_5_ and RNL_10_ compared to RNL_2.5_ and CN. Thus, it could be concluded that inclusion of *Rumex nepalensis* leaf powder in the diet resulted in improved performance and better immuno-antioxidant status of broilers. Further, an improvement in the gut health was observed in terms of positive effects on cecal microbiota and intestinal histomorphology of broiler chickens.

## 1. Introduction

The poultry industry in India has seen a quantum leap during last two decades mainly due to exploitation of modern growth promoting strategies and disease prevention measures [1,2]. Conventionally, antibiotic growth promoters (AGPs) have been used in poultry for many decades to enhance production and control pathogenic microbes [3,4,5,6,7,8,9]. However, irrational and indiscriminate use of AGPs resulted in the development of antibiotic-resistant bacteria [10,11,12], residues in animal products and probable transfer of resistant strains via the food chain to humans [13]. Due to these public health concerns and consumers pressure, AGPs were banned by the European Union in 2006 and China in 2020 [14] and researchers looked for other safe alternatives like phytogenics.

Phytogenic feed additives (PFAs) also called phytobiotics or botanicals are a group of natural growth promoters derived from herbs, spices, plants and their extracts like essential oils. PFAs are natural, less toxic, residue-free and ideal feed additives for poultry compared to synthetic antibiotics [15]. The main active components of phytobiotics include phenolics, tannins, glycosides and alkaloids [16]. Numerous studies have shown that PFAs in broiler diet improve digestive, intestinal functions and gut microflora [17], increase nitrogen retention and fiber digestibility [18], suppress inflammation [19] and increase antioxidative [20,21] antimicrobial activities [22], thereby improving the overall performance and broiler health.

*Rumex nepalensis* (Nepal Dock) phytogenic belonging to family Polygonaceae, is a perennial herb widely distributed throughout the Himalayas between 900–4000 m on moist as well as dry slopes [23]. The young shoots and juvenile leaves of this herb are cooked as vegetables [24,25]. Phytochemical screening of this herb shows various constituents, viz., triterpenoids, flavonoids, alkaloids, phenolic compounds, sterols, saponins, stilbene glycosides, anthraquinones, vitamin C, cardiac glycosides, naphthalenes [23,24]. In vitro and in vivo studies revealed that phytochemical compounds of phytogenics have antibacterial [26,27,28], antifungal [29,30,31], anticoccidial [32,33,34], immunomodulatory [35,36,37,38] activities. Due to lack of evidence regarding its utilization in the diet of poultry, the current study was taken up to evaluate the effect of feeding *Rumex nepalensis* leaf powder (RNL) on performance, blood markers, intestinal microbiology and histomorphology in broiler chickens.

## 2. Materials and Methods

### 2.1. Material

*Rumex nepalensis* herb was collected from various parts of Kashmir Valley, India. The identity of the herb was validated in the Department of Environmental Science, SKUAST-Kashmir, India. The leaves were separated and dried in a shade under sunlight. The dried leaves were ground into fine powder in the analytical laboratory of Division of LPM, FVSc & AH, SKUAST-Kashmir, India.

### 2.2. Proximate Composition

The proximate analysis of RNL for dry matter (DM), crude proteins (CP), ether extract (EE), crude fiber (CF), total phosphorous (P) and total ash (TA) was carried out as per the recommended methods of the AOAC [39] and calcium by Talpatra et al. [40]. The analysis was performed in triplicate and mean values were calculated.

### 2.3. Ethical Approval

The experiment was conducted in accordance with the guidelines of the Committee for the Purpose of Control and Supervision of Experiments on Animals (CPCSEA), Government of India, and approved (FVSc/VCC-9/20/IAEC/315-16) by the Institutional Ethics committee of Faculty of Veterinary Sciences, Shuhama, SKUAST-Kashmir. 

### 2.4. Broiler Chicks, Experimental Design and Management

A total of 180 day-old male VenCobb broiler chicks (average body weight 43 ± 1.8 g) were procured from a local commercial hatchery and transferred to the experimental unit of Division of LPM, FVSc & AH, SKUAST-Kashmir, India. The chicks were reared together for one week, weighed and randomly distributed to 12 floor pens (10 ft × 3 ft) having sawdust as litter material throughout the experiment (42 days). The experiment was conducted in a completely randomized design having four treatments with three replications (15 birds per replicate). Four iso-caloric and iso-nitrogenous diets primarily based on maize–soybean were formulated, viz., CN (Control)—fed basal diet only; RNL_2.5_ (basal diet + 2.5 g/kg RNL); RNL_5_ (basal diet + 5 g/kg RNL); and RNL_10_ (basal diet + 10 g/kg RNL). The basal diet fed to broiler chickens was formulated according to the guidelines of the NRC [41]. The ingredients and nutrient composition of the basal diet are given in Table 1. The birds were fed mash diets with ad libitum access to feed and fresh water. Vaccination schedule and bio-security measures were strictly followed and experiment was conducted under strict hygiene protocols. The temperature was kept at 33 °C and decreased by 3 °C every week till it reaches 24 °C. Birds were provided a photoperiod of 23 h light and 1 h dark till the end of experiment.

### 2.5. Measurement of Performance 

The body weight (BW) and feed intake (FI) per replicate were recorded weekly. Body weight gain (BWG) and FI were calculated for the starter (7–21 d), finisher (22–42 d) and entire period (7–42 d) of the experiment. Similarly, the feed conversion ratio (FCR) was calculated for the same periods by dividing the cumulative feed consumption to cumulative BWG and corrected for mortality if any. 

### 2.6. Sample Collection

At the end of experiment (42nd d), two birds per replicate were selected randomly and blood samples were collected in clot activator vacutainers from the wing vein. Each of the birds was then bled by Halal method in the slaughterhouse of the Division of Livestock Products Technology, FVSc & AH, SKUAST-Kashmir. Cecal contents were immediately collected into sterile polybags and stored at −20 °C for microbial count. Duodenum and jejunum (end of duodenum to Meckel’s diverticulum) samples (approximately 2 cm in length) were cut with scissors and washed in physiological saline to remove all the contents and then fixed in 10% buffered formalin solution for subsequent histomorphological investigations. 

### 2.7. Serum Biochemical Profile

The tubes containing blood were incubated at 37 °C for 1 h. Blood clots were shattered, and tubes were centrifuged at 3000 rpm for 30 min. The serum was pipetted out in small tubes and stored under deep freeze (−20 °C) until analysis. The parameters studied were total protein, glucose, cholesterol, alanine transaminase (ALT) and aspartate transaminase (AST) and creatinine with the aid of auto analyzer equipment using respective biochemical kits.

### 2.8. Immune and Antioxidant Parameters

The total serum immunoglobulin (IgG and Ig M) levels were determined using ELISA kits according to the methodology mentioned by the manufacturer (Shanghai, China). The serum enzymes superoxide dismutase (SOD) and malondialdehyde (MDA) were determined as per the methodology of Kakkar et al. [42] and Bradford [43], respectively.

### 2.9. Microbial Examination

For analysis of cecal microbiology, one gram of each cecal collected sample was homogenized in 9 mL of sterilized saline peptone solution and stirred for 30 min to obtain 10^−1^ dilution. Serial 10-fold dilutions were made up to 10^−6^ in pre-sterilized tubes. Readymade media from Hi-media Laboratories Pvt. Ltd., Mumbai, was used for growth of different microbes. An aliquot of 0.1 mL of each dilution was spread over different specific media such as plate count agar (PCA) for total bacterial count (TBC) after incubation at 30 °C for 48 h, MacConkey agar for coliforms (37 °C for 24 h) and DeMan–Rogosa–Sharpe (MRS) agar for lactic acid bacteria (37 °C for 48 h). Total plate count (TPC), coliform and *Lactobacillus* counts in the cecal contents were enumerated as per the method described by Speck [44]. Duplicate plates were prepared and the counts were expressed as log10 colony forming units per gram (cfu/g).

### 2.10. Histomorphometric Study

The samples of duodenum and jejunum fixed in 10% formalin were dehydrated and embedded in paraffin wax. Then, sections (6 µm thick) were cut out of the paraffin-embedded tissue blocks using a microtome and stained with hematoxylin and eosin. Stained tissues were examined under a microscope (Nikon Eclipse Ni, DS-Riz, Melville, NY, USA), equipped with a computer assisted morphometric system. Villus height (VH) and crypt depth (CD) were measured as the mean of 10 randomly selected parts in each sample. The VH to CD ratio was then calculated accordingly [45]. 

### 2.11. Statistical Analysis

Data were analyzed using one-way ANOVA. The statistical model used was:

*Y_ij_* = *µ* + *T_i_* + *e_ij_*

where *Y_ij_* represents the observation for the dependent variables at the jth replicate in the ith treatment (*i* = 1 to 4), µ is the overall mean, *T_i_* is the fixed effect of treatments (*i* = 1 to 4) and *e_ij_* is the random error. 

The means were compared using Duncan’s multiple range test. Each replicate served as the experimental unit for comparing growth performance, whereas birds selected per replicate served as the experimental units for the remaining parameters. The significance was declared at *p* < 0.05.

## 3. Results

### 3.1. Proximate Composition of RNL

The proximate analysis of RNL revealed 22.46% CP, 3.28% EE, 19.70% CF, 5.84% TA, 0.86% Ca and 0.47% P on % DM basis.

### 3.2. Growth Performance

The results pertaining to BWG, FI and FCR are presented in Table 2. During the starter (7–21 d), finisher (22–42 d) and entire experimental (7–42 d) period, BWG and FCR improved in dietary treatments when compared to CN. RNL_5_ and RNL_10_ had significantly (*p* < 0.05) higher BWG and decreased than RNL_2.5_ and CN. However, FI did not vary (*p* > 0.05) among dietary treatments and CN.

### 3.3. Serum Biochemistry

Table 3 shows the results of blood markers in broiler chickens at 42nd day of age. Supplementation of RNL did not produce significance (*p* > 0.05) in total protein, glucose, creatinine, AST and ALT levels among all the treatments including CN. However, dietary treatments caused a significant (*p* < 0.05) reduction in the serum cholesterol levels compared to CN with peak reduction in the RNL_10_ group.

### 3.4. Immune and Antioxidant Study

The serum immune and antioxidant results are depicted in Table 4. A dose-dependent increase in the serum IgG and IgM levels was observed with the increase of the dose of *Rumex nepalensis* in the diet. The highest improvement in the IgG and IgM concentration was recorded in RNL_10_. Further, the serum SOD levels increased and MDA levels decreased significantly in RNL_10_ when compared with all other dietary treatments and control.

### 3.5. Microbial Enumeration

Cecal microbial enumeration of broiler chickens at 42nd day of age is presented in Table 5. A remarkable decrease in cecal coliform count was observed in all the dietary treatments supplemented with RLP with respect to CN. The maximum decrease (*p* < 0.05) in the coliform count was observed in the RNL_10_ group followed by RNL_5_. The LAB count and TPC did not differ significantly (*p* > 0.05) between dietary treatments and CN.

### 3.6. Duodenal and Jejunal Histomorphometry

**Figure 1:**
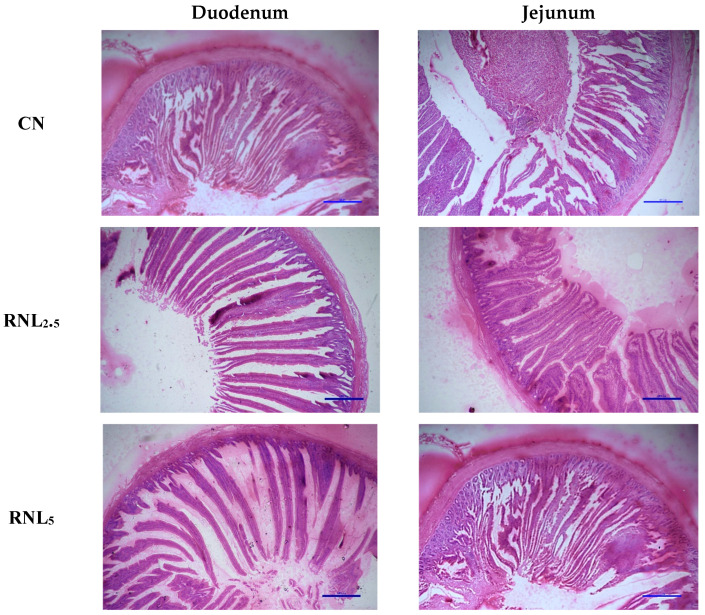

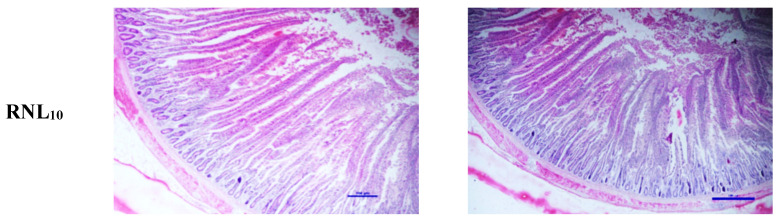
Representative histological micrographs of duodenum and jejunum in broiler chickens on day 42. Scale bar indicates 500 µm. CN (Control)—fed basal diet only; RNL_2.5_ (basal diet + 2.5 g/kg *Rumex nepalensis* leaf powder); RNL_5_ (5 g/kg *Rumex nepalensis* leaf powder); RNL_10_ (10 g/kg *Rumex nepalensis* leaf powder).

The duodenal and jejuna histomorphological alterations of broiler chickens in various treatments at 42 days of age are shown in Figure 1 and Table 6. Both duodenal and jejunal VH increased significantly (*p* < 0.05) in the RNL_5_ and RNL_10_ groups compared to CN. No significance (*p* > 0.05) was observed in CD values between CN and various dietary treatments. The VH/CD ratio was significantly (*p* < 0.05) improved in all the treatments supplemented with RLP with the highest values in RNL_5_ and RNL_10_.

## 4. Discussion

The proximate analysis of RNL revealed 22.46% CP, 3.28% EE, 19.70% CF, 5.84% TA, 0.86% Ca and 0.47% P on % DM basis. Azzam et al. [46] investigated leaf powder of another species of *Rumex,* viz., *Rumex nervosus,* and reported CP of 13.63%, CF (8.24%), EE (1.54%) and TA (18.01%). The variation in the results might be due to the different species of *Rumex* studied in the present investigation.

In our study, the RNL_5_ and RNL_10_ groups showed improved BWG and FCR in broiler chickens compared to CN. These results could be attributed to the presence of various bioactive compounds in *Rumex nepalensis* such as triterpenoids, flavonoids, alkaloids, phenolic compounds, sterols, saponins, stilbene glycosides, anthraquinones, vitamin C, cardiac glycosides, naphthalenes etc. [23,24]. Numerous studies have documented that phytogenic addition in broiler diets with garlic @ 0.5–2% [47], ginger @ 1% [48], peppermint @ 70 mg/kg [49], thyme @ 0.01–0.03% [50], had positive effects on BWG and FCR. The beneficial effects have been attributed to the fact that phytogenics stimulate saliva production, secretion of digestible enzymes, resulting in increased digestion and utilization of nutrients in the gut [51,52]. Phytogenics have been shown to decrease pathogenic bacteria [53] and increase beneficial microflora like *Lactobacilli*, which protect the bird from various stresses and increase its performance [54,55]. Other effects include positive influence on the intestinal architecture in terms of increasing the villus height (VH), resulting in increased surface area for absorption of nutrients and more mucus secretion [56,57,58]. However, a few reports have shown inconsistent effects of phytogenics on growth performance of birds [59,60]. No biological study has been carried out so far regarding utilization of *Rumex nepalensis* in the diet of broiler chicken, though a few studies have been conducted on other species, i.e., *Rumex nervosus*. As reported by Azzam et al. [46], a significant improvement in BWG and FCR was observed during the 1–14 d period by addition of *Rumex nervosus* leaf powder up to 5 g/kg in broiler diet, which supports the results obtained in the present study. Similar to our results, Alqhtani et al. [61] reported an improvement in BWG during the overall period (1–34 d) on supplementation of *Rumex nervosus* leaf powder up to 5 g/kg in the broiler diet. 

The serum biochemical profile is one of the indicators of physiological, nutritional and pathological status of a bird. So, while screening a therapeutic compound, the serum biochemical profile can be correlated to identify its impact, which will determine its compatibility for a particular species [62,63]. The serum biochemical profile of a bird changes with age and during certain conditions like nutrition, diseases, etc. The serum total protein levels obtained in our study revealed no significant difference between control and various treatments and the results were well within the normal range (2.5–4.5 g/dL) [64]. These results are in agreement with the values as reported by Alqhtani et al. [61]. The serum total protein levels depict the protein reserves in the body [65]. Proteins are required for growth and various other functions like immunity and disease prevention and low levels indicate liver damage or other diseases. In contrast, a decrease in the level of serum protein was reported by addition of *Rumex nervosus* leaves in the diet of broiler chicken [66]. A numerical decrease in the serum glucose levels was observed in the treatments compared to control, which do not reach statistical significance and were within or near normal range [67]. However, Alqhtani et al. [61] reported a significant decrease in the serum glucose level on supplementation of *Rumex nervosus* leaf powder at 1 g/kg diet compared to control, but no significance was observed at 5 g/kg diet. Total cholesterol levels varied significantly between the control and various treatments. In the present study, the lowest levels were observed in RNL_10_ followed by RNL_5_, which might be due to various bioactive compounds present in *Rumex nepalensis*. However, contrary to our results, Alqhtani et al. [61] reported a non-significant decrease in the cholesterol on supplementation of *Rumex nervosus* leaf powder up to 5 g/kg diet compared to control. The activity of the enzymes ALT and AST is an important indicator of a healthy liver [68]. The increase in the concentration of these enzymes in the serum reflects liver damage or a malfunction [69]. Our study revealed no significance in both ALT and AST, which agrees with the results obtained by Azzam et al. [46] on supplementation of *Rumex nervosus* leaf powder up to 5 g/kg diet, but contrary to our results, Alqhtani et al. [61] reported a significant decrease in the activity of ALT compared to control. Kidneys are vital organs of excretion which relieve the body from waste products resulting from various metabolic processes and muscles contraction [70]. Serum creatinine level is one of the most sensitive biochemical markers employed in the diagnosis of renal damage [71]. An increase in the serum creatinine levels beyond normal range depicts kidney damage. The values of serum creatinine obtained in our study revealed no significance (*p* > 0.05) on supplementation of RNL up to 10 g/kg diet compared to control, which is in agreement with the values as reported by Alqhtani et al. [61].

The serum concentration of IgG and IgM was significantly increased by supplementation of *Rumex nepalensis* @ 5 and 10 g/kg diet. This indicated immune modulatory effect of supplementation of *Rumex nepalensis* in broiler chickens. Phytogenic compounds may induce their immunomodulatory effects through increasing immune cells proliferation, arising cytokines expression and elevation of antibody titers [36]. SOD protects the intracellular lipids against peroxidation while MDA is an indication of lipid peroxidation level [72]. The higher the levels of SOD and the lower the levels of MDA, the better is the antioxidant status of the bird. *Rumex* has a high level of antioxidant enzymes and scavenges free radicals in order to protect the lipid peroxidation of the intestinal mucosa and cells [73]. In the present study, a significant increase in SOD and decrease in MDA was noticed in the group fed 10 g *Rumex nepalensis*/kg of diet, indicating its antioxidant effect in broiler birds. 

Poultry health is closely associated with gut microbiome composition and diversity. The gut microbiota of the GIT include both beneficial bacteria (*Lactobacilli* and *Bifidobacteria*) and pathogenic bacteria (*E.coli*, *Campylobacter* and *Salmonella*) [74]. The microbiome of the GIT is involved in protection against pathogens, nutrient production and absorption, maturation of the immune system and the overall performance of birds [75]. A poor gut health results in malabsorption of nutrients and reduced growth in birds [76]. Various studies in broilers revealed that indiscriminate use of AGPs led to imbalance in the host gut microbiome by killing desirable bacteria like lactobacilli [77]. Moreover, in a recent study, an improvement in the diversity of gene families involved in the degradation of cellulose, hemicellulose and starch was observed in chickens that did not receive AGPs [78]. Phytogenic compounds are known to regulate the gut microflora and thereby help in maintaining the host health [79]. Numerous studies have shown that phytogenic compounds or their extracts decrease the population of pathogenic microbes and their metabolites and increase the growth of beneficial microflora, which protects the bird against various infections and improves their performance [55]. Our study revealed a significant reduction in the coliform count in *Rumex*-supplemented groups compared to control. However, TPC and lactobacilli count did not vary between control and dietary treatments. These results are in agreement with Azzam et al. [46], who reported a significant reduction in cecal *E. coli* count on supplementation of *Rumex nervosus* leaf powder up to 3 g/kg diet, with no change in the lactobacilli count. Phytogenics have been shown to reduce total viable count and coliform count which favor the growth of beneficial microflora like lactobacilli, thereby protecting poultry birds from various infections and improve their overall performance [80]. 

The absorption capacity of the small intestine can be shown by the morphometry of villus and crypts and the VH/CD ratio [81]. There exists a dynamic equilibrium between the production of enterocytes in the crypts and their subsequent desquamation from the villus, so the ratio of VH/CD can be a criterion to evaluate healthy and functioning intestines [82]. Longer villi with a greater surface area can lead to better feed utilization, improved performance and health of birds [83]. Phytogenics has been observed to improve the micro-architecture of the intestines by increasing the villus height and surface area [84], absorption capacity of dendritic cells and stimulating mucus secretion, consequently easing pathogen adhesion in the intestines [48]. Oladele et al. [85] reported an improvement in VH, width and CD in broiler diets supplemented with 0.125% garlic meal. Karangiya et al. [58] found that addition of ginger at 1% to broiler diet significantly increased villi length, width and cryptal depth and consequently increased surface area for absorption of nutrients. In our study, *Rumex* supplementation increased VH and VH/CD ratio in both duodenum and jejunum compared to control. The results of our study were partly in agreement with the previous workers [46] who reported an increase in VH and VH/CD ratio in broilers supplemented with *Rumex nervosus* leaf powder up to 3 g/kg diet, but at 5 g/kg diet no significance was observed compared to control. The increase in VH and VH/CD ratio across the small intestine increases the surface area for absorption of nutrients which partly explains the better performance observed in our study.

## 5. Conclusions

Inclusion of a tested batch of *Rumex nepalensis* leaf powder in the diet resulted in improved performance of broiler birds in terms of better weight gain and feed efficiency. Further, addition of dietary *Rumex nepalensis* leaf powder had cholesterol lowering effect in the birds, besides enhancing immuno-antioxidant status. Moreover, supplementation of *Rumex nepalensis* leaf powder in the feed of broilers showed beneficial impact on gut health in terms of modifying cecal microbiota and intestinal histomorphology in a positive manner. However, further studies are warranted in order to optimize the proper dosage of adding *Rumex nepalensis* leaf powder as a feed additive in broilers. 

## Figures and Tables

**Table 1:** Ingredients and nutrient composition of broiler chicken diets.

Ingredients (g/kg)	Starter (7–21 Day)	Finisher (22–42 Day)
Maize (9.5%) *	55.98	60.45
Soybean meal (48%) *	34.00	24.70
Fish meal (55%) *	4.00	3.00
Mustard oil	3.00	3.50
Limestone	0.80	0.80
Di-calcium phosphate	1.40	1.60
Salt	0.30	0.30
DL-Methionine	0.11	0.10
Lysine	0.06	0.20
Trace mineral Premix ^1^	0.10	0.10
Vitamin Premix ^2^	0.15	0.15
Choline chloride	0.05	0.05
Toxin binder	0.05	0.05
Total	100	100
Analyzed nutrient		
Crude Protein (%)	21.93	20.01
Calculated nutrient		
Metabolizable Energy (Kcal/kg)	3052.43	3138.25
Calcium	1.01	1.00
Available P	0.46	0.45
Lysine	1.22	1.13
Methionine	0.49	0.49

^1^ Trace mineral premix (mg/kg diet): Mg 300, Mn 55, I 0.4, Fe 56, Zn 30 and Cu 4. ^2^ Vitamin premix (per kg diet): Vitamin A 8250 IU, Vitamin D3 200 IU, Vitamin K 1 mg, Vitamin E 40 IU, Vitamin B1 2 mg, Vitamin B2 4 mg, Vitamin B6 4 mg, Biotin 0.2 mg, Folic acid 1 mg, Vitamin B12 10 µg, Niacin 60 mg, Pantothenic acid 10 mg, choline, 500 mg. * Indicates CP percentage of individual ingredients.

**Table 2:** Effect of supplementation of *Rumex* on performance of broiler chickens.

Parameter	Dietary Treatments ^1^				SEM	*p*-Value
	CN	RNL_2.5_	RNL_5_	RNL_10_		
BWG, grams						
7–21 d	456 ^b^	479 ^ab^	487 ^a^	494 ^a^	5.66	*p* < 0.05
22–42 d	1303 ^b^	1315 ^ab^	1347 ^a^	1354 ^a^	8.09	*p* < 0.05
7–42 d	1759 ^b^	1794 ^ab^	1837 ^a^	1848 ^a^	13.43	*p* < 0.05
FI, grams						
7–21 d	568	575	582	586	5.04	*p* > 0.05
22–42 d	2506	2509	2504	2509	6.71	*p* > 0.05
7–42 d	3075	3084	3086	3094	9.03	*p* > 0.05
FCR						
7–21 d	1.25 ^a^	1.20 ^b^	1.20 ^b^	1.19 ^b^	0.009	*p* < 0.05
22–42 d	1.92 ^a^	1.91 ^a^	1.86 ^b^	1.85 ^b^	0.010	*p* < 0.05
7–42 d	1.75 ^a^	1.72 ^a^	1.68 ^b^	1.68 ^b^	0.009	*p* < 0.05

^1^ CN (Control)—Basal diet only; RNL_2.5_—Basal diet + 2.5 g/kg *Rumex*; RNL_5_—Basal diet + 5 g/kg *Rumex*; RNL_10_—Basal diet + 10 g/kg *Rumex*. ^ab^ Means within a row bearing different superscripts differ significantly (*p* < 0.05). BWG—Body weight gain; FI—Feed intake, FCR—Feed conversion ratio.

**Table 3:** Effect of supplementation of *Rumex* on serum biochemical profile of broiler chickens.

Parameter	Dietary Treatments ^1^				SEM	*p*-Value
	CN	RNL_2.5_	RNL_5_	RNL_10_		
Total Protein (g/dL)	3.37	3.29	3.46	3.51	0.06	*p* > 0.05
Glucose (mg/dL)	225.89	215.14	205.41	196.37	6.74	*p* > 0.05
Cholesterol (mg/dL)	143.74 ^a^	126.82 ^ab^	115.79 ^b^	103.25 ^b^	5.41	*p* < 0.05
ALT (U/L)	19.30	18.27	16.15	15.02	1.22	*p* > 0.05
AST (U/L)	144.78	141.19	137.74	131.30	4.99	*p* > 0.05
Creatinine (mg/dL)	0.62	0.64	0.60	0.72	0.05	*p* > 0.05

^1^ CN (Control)—Basal diet only; RNL_2.5_—Basal diet + 2.5 g/kg *Rumex*; RNL_5_—Basal diet + 5 g/kg *Rumex*; RNL_10_—Basal diet + 10 g/kg *Rumex*. ^ab^ Means within a row bearing different superscripts differ significantly (*p* < 0.05). ALT—Alanine aminotransferase; AST—Aspartate aminotransferase.

**Table 4:** Effect of supplementation of *Rumex* on immune and antioxidant parameters of broiler chickens.

Parameter	Dietary Treatments ^1^				SEM	*p*-Value
	CN	RNL_2.5_	RNL_5_	RNL_10_		
IgG (µg/mL)	277.40 ^c^	292.93 ^bc^	315.62 ^ab^	332.64 ^a^	6.64	*p* < 0.05
IgM (µg/mL)	529.74 ^b^	541.45 ^b^	553.92 ^ab^	577.09 ^a^	5.83	*p* < 0.05
SOD (U/mL)	132.07 ^b^	135.46 ^b^	141.63 ^b^	155.88 ^a^	3.05	*p* < 0.05
MDA (nmol/mL)	7.11 ^a^	7.02 ^ab^	6.90 ^bc^	6.82 ^c^	0.04	*p* < 0.05

^1^ CN (Control)—Basal diet only; RNL_2.5_—Basal diet + 2.5 g/kg *Rumex*; RNL_5_—Basal diet + 5 g/kg *Rumex*; RNL_10_—Basal diet + 10 g/kg *Rumex*. ^abc^ Means within a row bearing different superscripts differ significantly (*p* < 0.05). IgG—Immunoglobulin G, IgM—Immunoglobulin M, SOD—Superoxide dismutase, MDA—Malondialdehyde.

**Table 5:** Effect of supplementation of *Rumex* on cecal microbial count of broiler chickens.

Parameter (cfu/g)	Dietary Treatments ^1^				SEM	*p*-Value
	CN	RNL_2.5_	RNL_5_	RNL_10_		
Coliform count	7.16 ^a^	6.84 ^ab^	6.78 ^ab^	6.57 ^b^	0.080	*p* < 0.05
Lactobacilli count	5.98	6.04	6.07	6.11	0.083	*p* > 0.05
TPC	8.93	8.75	8.84	8.71	0.064	*p* > 0.05

^1^ CN (Control)—Basal diet only; RNL_2.5_—Basal diet + 2.5 g/kg *Rumex*; RNL_5_—Basal diet + 5 g/kg *Rumex*; RNL_10_—Basal diet + 10 g/kg *Rumex*. ^ab^ Means within a row bearing different superscripts differ significantly (*p* < 0.05). TPC—Total plate count.

**Table 6:** Effect of supplementation of *Rumex* on intestinal morphometry of broiler chickens.

Parameter	Dietary Treatments ^1^				SEM	*p*-Value
	CN	RNL_2.5_	RNL_5_	RNL_10_		
Duodenum						
Villus height (µm)	1342 ^c^	1367 ^bc^	1383 ^ab^	1396 ^a^	6.95	*p* < 0.05
Crypt depth (µm)	195	186	179	174	3.44	*p* > 0.05
VH/CD ratio	6.89 ^b^	7.36 ^ab^	7.76 ^a^	8.02 ^a^	0.16	*p* < 0.05
Jejunum						
Villus height (µm)	1204 ^b^	1230 ^ab^	1247 ^a^	1256 ^a^	7.02	*p* < 0.05
Crypt depth(µm)	198	191	182	179	3.27	*p* > 0.05
VH/CD ratio	6.09 ^b^	6.46 ^ab^	6.85 ^a^	7.04 ^a^	0.14	*p* < 0.05

^1^ CN (Control)—Basal diet only; RNL_2.5_—Basal diet + 2.5 g/kg *Rumex*; RNL_5_—Basal diet + 5 g/kg *Rumex*; RNL_10_—Basal diet + 10 g/kg *Rumex*. ^abc^ Means within a row bearing different superscripts differ significantly (*p* < 0.05).

## Data Availability

All the data presented in the study are included in the article; further inquiries can be directed to the corresponding authors.
